# Sleep disturbances, inflammation, and muscle function in older adults with sarcopenia

**DOI:** 10.1007/s11325-026-03795-9

**Published:** 2026-09-04

**Authors:** Helton de Sá Souza, Luciano Bernardes Leite, Pedro Forte, Pedro Henrique Viana Mendes, Guilherme Pereira Saborosa, Rafael Eduardo Eustórgio Pinheiro Chagas Miranda, Leoncio Lopes Soares, Ronaldo Vagner Thomatieli-Santos, Bruno Moreira Silva, Sergio Tufik, Vânia D’Almeida

**Affiliations:** 1 Present Address: Department of Physical Education, Universidade Federal de Viçosa, Av. P. H. Rolfs, Minas Gerais 36570-000 Viçosa, Brazil; 2 Department of Sports, Instituto Politécnico de Bragança, Bragança, 5300-253 Portugal; 3 Department of Sports, Higher Institute of Educational Sciences of the Douro, Penafiel, 4560-708 Portugal; 4 CI-ISCE, Instituto Superior de Ciências Educativas do Douro (ISCE Douro), Penafiel, 4560-547 Portugal; 5 Research Center for Active Living and Wellbeing (LiveWell), Instituto Politécnico de Bragança, Bragança, 5300-253 Portugal; 6 Physiotherapy and Pain Group, Department of Physical Therapy, University of Alcala, Madrid, 28801 Spain; 7 Department of Psychobiology, Universidade Federal de São Paulo, São Paulo, 04023-062 SP Brazil; 8 Department of Physical Education, Universidade Federal de Juiz de Fora, MG Governador Valadares, Brazil; 9 Department of Biosciences, Universidade Federal de São Paulo, Santos, 11015-020 SP Brazil; 10 Department of Physiology, Universidade Federal de São Paulo, São Paulo, 04023-062 SP Brazil

**Keywords:** Sleep efficiency, Cytokines, Handgrip strength, Physical performance, Muscle catabolism

## Abstract

**Introduction:**

Sarcopenia, a geriatric syndrome of muscle and functional loss that increases the risks of falls and mortality, may be aggravated by sleep disturbances that impair protein synthesis and promote catabolism via inflammation. The present study aimed to investigate the association between sleep patterns, inflammatory markers, and muscle function in older adults with and without sarcopenia.

**Methods:**

This prospective controlled study included 63 older adults (> 65 years), classified into sarcopenic (ES, *n* = 31) and non-sarcopenic (ENS, *n* = 32) groups. Assessments included body composition, muscle function, inflammatory biomarkers, mood, and sleep (polysomnography, actigraphy, questionnaires).

**Results:**

The ES showed lower handgrip strength (21.7 ± 7.6 vs. 27.6 ± 10.4 kg; *p* < 0.05) and poorer physical performance (SPPB: 8.8 ± 2.3 vs. 11.2 ± 1.0; *p* < 0.05). They presented reduced thigh extension torque (82.7 ± 35.1 vs. 113.0 ± 26.8 N·m; *p* < 0.05) and flexion torque (44.4 ± 23.7 vs. 53.3 ± 16.0 N·m; *p* < 0.05). Sleep alterations included shorter total sleep time (*p* < 0.05), lower sleep efficiency (*p* < 0.05), higher WASO (*p* < 0.05), and increased apnea-hypopnea index (*p* < 0.05). TNF-α levels were higher in ES (*p* < 0.05). Mood disturbances were also greater, with lower vigor (*p* < 0.05) and higher TMD (*p* < 0.05).

**Conclusion:**

Sarcopenic older adults exhibit muscle impairments, sleep disturbances, and increased inflammation, which may accelerate sarcopenia progression, making sleep and inflammation important targets for intervention.

## Introduction

Sarcopenia, a geriatric syndrome characterized by the progressive and generalized loss of muscle mass, strength, and function, has emerged as a growing global public health concern [1, 2]. Its prevalence increases significantly with aging, leading to adverse outcomes such as elevated risk of falls, frequent hospitalization, and increased mortality among older adults [2–5]. The pathogenesis of sarcopenia is multifactorial, involving complex factors such as hormonal dysregulation, chronic low-grade inflammation, and physical inactivity [2]. Recently, research has highlighted sleep disturbances as potential modulators of sarcopenia risk and progression. Sleep deprivation and fragmentation have been associated with detrimental effects on muscle health, potentially accelerating the decline in muscle mass and function [6, 7].

Recent evidence also highlights a biological link between sleep regulation, inflammation, and muscle metabolism [8–10]. Disturbances in the sleep–wake cycle can disrupt circadian control of muscle protein synthesis, impairing anabolic signaling pathways involved in repair and hypertrophy [8, 11]. Sleep disturbances have been increasingly linked to alterations in inflammatory and myokine regulation, which play important roles in metabolic and muscle homeostasis [12–14]. In parallel, circadian rhythm disruptions have been associated with changes in the expression of genes involved in muscle protein synthesis, energy metabolism, and mitochondrial function, suggesting a physiological pathway through which poor sleep may contribute to muscle decline [11, 15, 16].

Sleep plays a crucial role in muscle recovery and the regulation of energy metabolism [17]. Growing evidence shows that reduced sleep duration and quality are correlated with decreased protein synthesis, increased muscle catabolism, and poorer physical performance outcomes in the elderly [6]. Furthermore, common conditions among older adults (i.e., such as obstructive sleep apnea) are known to induce a pro-inflammatory state, which may exacerbate or even contribute to the development of sarcopenia [18]. Despite these emerging associations, there remains a notable gap in the literature regarding detailed studies exploring the relationship between subjective aspects of sleep (i.e., such as perceived quality and duration) and sarcopenia, particularly in older individuals already experiencing some degree of muscle impairment.

Systemic inflammation emerges as a critical link in this complex interplay, being frequently associated with both muscle loss and sleep disturbances [19]. Elevated levels of pro-inflammatory cytokines, such as tumor necrosis factor-alpha (TNF-α), have been linked to reduced muscle strength and increased daytime sleepiness, negatively impacting the quality of life in older adults [20]. Conversely, alterations in sleep patterns can impair physical performance; reductions in total sleep time (TST) and poor sleep quality may hinder muscle recovery and mobility [21]. Understanding the intricate relationship among sleep disturbances, inflammation, and muscle function is therefore imperative for developing effective strategies to prevent and manage sarcopenia in the aging population. In this context, the present study aims to compare sleep patterns, inflammatory markers, and physical performance between older adults with and without sarcopenia. Our objective is to investigate whether differences in sleep patterns and systemic inflammation are intrinsically linked to muscle impairment in this population, providing critical insights for future clinical interventions.

## Materials and methods

### Study design and participants

This was a prospective, single-center study conducted at the Federal University of São Paulo/Hospital São Paulo (UNIFESP/HSP). The study was approved by the Research Ethics Committee (CEAA: 51594215.9.0000.5505). Participants were community-dwelling older adults aged over 65 years, of any sex, with controlled metabolic or osteoarticular conditions. Exclusion criteria included individuals with unstable clinical conditions; a history of cancer, cardiovascular diseases, or chronic obstructive pulmonary disease; disabling psychiatric disorders; or the use of GLP-1 inhibitors and medications that could influence the sleep-wake cycle.

Following approval from the ethics committee, the study was disseminated through major newspapers, television channels, radio stations, and the social media platforms of the Federal University of São Paulo and the researchers involved in the project. A total of 358 older adults were initially contacted by telephone, during which personal data, medical history, and lifestyle habits were collected. Of these, 257 declined participation and 101 attended the in-person screening visit. After application of the eligibility criteria, including exclusion of individuals with predefined clinical conditions and lifestyle characteristics, 63 participants completed the baseline assessments and were included in the present study. Eligible individuals attended an in-person visit to sign the Free and Informed Consent Form (FICF), followed by the administration of questionnaires and physical performance tests, handgrip strength assessment, body composition evaluation, blood sample collection, and actigraph placement. After 10 days of actigraph use, participants returned to the laboratory for overnight polysomnography. 

### Clinical assessment and diagnosis of sarcopenia 

The diagnosis of sarcopenia was carried out by a multidisciplinary team composed of a geriatrician and a sports medicine physician, both with expertise in the assessment of older adults. The diagnostic methodology strictly followed the criteria established by the European Working Group on Sarcopenia in Older People (EWGSOP) [22, 26], a widely recognized consensus in geriatric literature. Although a more recent consensus has emerged, the data collection took place before its publication, and for this reason, the consensus above was used. Sarcopenia was identified by the presence of reduced muscle mass in combination with either low muscle strength and/or impaired physical performance, according to the following criteria: muscle mass, assessed using the Appendicular Skeletal Muscle Mass Index (ASMI), with cut-off values of <7.27 kg/m² for men and <5.60 kg/m² for women; muscle strength, measured by handgrip strength using a dynamometer, with cut-off values of <40 kg for men and <30 kg for women; and physical performance, evaluated using the Short Physical Performance Battery (SPPB), with scores <6 points indicating poor performance. 

**Fig. 1:**
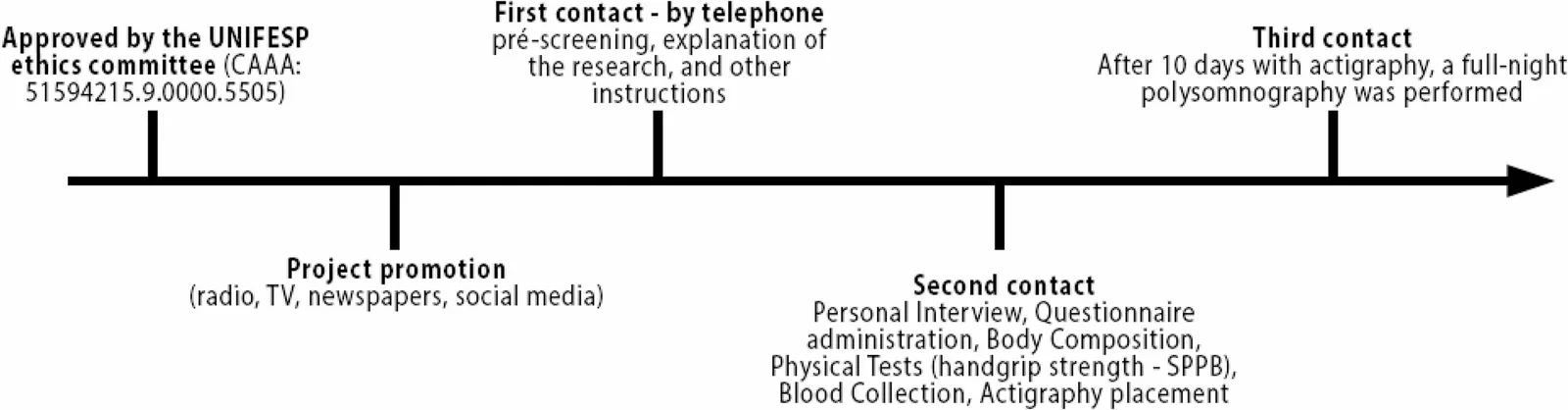
Timeline of data collection

Figure 1 Timeline of data collection. Following ethics committee approval, the project was publicized, and during the initial contact, potential participants were informed about the entire experimental protocol. Subsequently, eligible participants underwent a general clinical screening, a blood draw (between 7:00 AM and 10:00 AM), body composition assessments, physical tests, and questionnaire administration (PSQI, ISI, Horne & Östberg, GDS, STAI, and BRUMS). During the third contact, actigraphs were fitted to the participants, and fullnight polysomnography (PSG) was performed; electrode placement took place between 7:00 PM and 8:00 PM.

### Body composition assessment

Body composition was accurately assessed using Dual-Energy X-ray Absorptiometry (DXA), with the Prodigy device from GE-Lunar (Madison, WI, USA). Muscle mass and ASMI were determined from DXA data processed according to the manufacturer’s guidelines [23]. Participant height was measured using a wall-mounted stadiometer to ensure standardization, and body mass was recorded using a properly calibrated digital scale, following standard anthropometric measurement protocols.

### Physical function assessment

Physical function was thoroughly assessed using standardized tests. The Short Physical Performance Battery (SPPB) was applied, comprising balance tests, gait speed, and chair stand tests, providing a composite score that reflects overall functional performance. Additionally, the Timed Up and Go (TUG) test was used to evaluate functional mobility and fall risk. Muscle strength was measured through handgrip dynamometry (Jamar, Sammons Preston, USA), isokinetic dynamometry using the Biodex System 3 (Biodex Medical Systems, USA).

For isokinetic and isometric assessments, peak torque was used as an index of muscle function. Following a 5-minute warm-up on a cycle ergometer, participants performed three sets of four maximal concentric knee extension and flexion repetitions at an angular velocity of 60°/s, with 30-second rest intervals between sets. Isometric torque was assessed one hour later with the knee positioned at 90° of flexion, consisting of three maximal 10-second contractions. Participants were instructed and verbally encouraged to exert maximal effort during all tests. 

### Biochemical and hormonal assessments

Blood collection was performed between 7:00 and 10:00 a.m. at second contact to analyze the complete blood count (using ADVIA^®^ 360, Siemens Healthcare, Germany), lipid profile (Beckman Coulter Inc.), fasting blood glucose (Beckman Coulter Inc.), urea, creatinine, serum albumin, and 25-hydroxy vitamin D. Anabolic biomarkers, including testosterone, growth hormone (GH), and insulin-like growth factor 1 (IGF-1), along with the catabolic biomarker cortisol, were measured using the Chemiflex™ chemiluminescent immunoassay (Abbott Ireland Diagnostics Division, Ireland). Additionally, the inflammatory profile TNF-α, IL-6, IL-10, and IL-1ra was measured using ELISA (RayBio^®^, USA). These cytokines were selected because, together, they provide an overview of both pro and anti-inflammatory activity. 

### Objective sleep assessment

Sleep was evaluated using full-night polysomnography (Embla^®^ N7000, Embla Systems, USA) at the Sleep Institute – AFIP. This assessment included electroencephalography (EEG), bilateral submental and tibial electromyography, electrooculogram, electrocardiography, and respiratory measurements. All overnight polysomnography (PSG) assessments were conducted under a highly standardized protocol at the sleep laboratory, which features a maximum capacity of 13 simultaneous recordings per night. Upon arrival, all participants underwent electrode and sensor montage between 19:00 and 20:00, strictly following their final evening meal. The setup was performed by a team of experienced, certified sleep technicians. To maintain consistency while respecting individual bedtime habits, ‘lights-out’ was standardly scheduled at approximately 22:00; however, participants were permitted to initiate their sleep window immediately following the completion of the equipment setup if requested. all polysomnography (PSG) records were visually scored by certified sleep technicians following the standard criteria established by the American Academy of Sleep Medicine (AASM) [24]. Hypopnea was defined as a ≥ 30% reduction in airflow lasting for at least 10 s, associated with either a ≥ 3% reduction in oxygen saturation or an arousal. The apnea-hypopnea index (AHI) was calculated as the total number of apneas and hypopneas per hour of sleep. Polysomnographic parameters were analyzed as continuous variables and summarized at the group level as mean ± standard deviation. Individual polysomnographic results were used to derive the summary values presented in Table 4. The AHI was compared as a continuous measure of sleep-disordered breathing between the sarcopenic and non-sarcopenic groups [24]. Additionally, the sleep-wake cycle was monitored through actimetry (Motionlogger, Ambulatory Monitoring, USA) over a period of 10 consecutive days.

### Subbjective sleep assessment

The following validated questionnaires were employed in the study: the Pittsburgh Sleep Quality Index (PSQI), which evaluates subjective sleep quality, sleep latency, duration, and efficiency; the Epworth Sleepiness Scale, designed to assess the propensity for daytime sleepiness across different situations; the Insomnia Severity Index (ISI), which measures the severity and impact of insomnia symptoms; and the Horne and Östberg Chronotype Questionnaire, used to assess participants’ self-reported morningness-eveningness preference. The Horne-Östberg Morningness-Eveningness Questionnaire was administered to provide a complementary characterization of participants’ chronotype and sleep–wake preferences, contributing to a more comprehensive characterization of their sleep profile. 

### Mood and anxiety assessments

The following questionnaires were administered: the Geriatric Depression Scale (GDS) for assessing depressive symptoms, the State-Trait Anxiety Inventory (STAI) for measuring state and trait anxiety, and the Brunel Mood Scale (BRUMS) for analyzing six emotional states: tension, depression, anger, vigor, fatigue, and mental confusion. To minimize potential circadian influences on mood perception, all questionnaires were administered at the same time of day.

### Statistical Analysis

The sample size was determined using G*Power 3.1.7 software (Düsseldorf, Germany), with an expected effect size of 0.25%, a significance level of *p* ≤ 0.05, and a statistical power of 95%. Given the final number of participants, the study was not powered to perform reliable sex- or BMI-stratified analyses, and therefore moderating effects of these variables could not be explored. This calculation resulted in a total sample of 64 volunteers, divided equally into two groups: 32 with sarcopenia and 32 without.

Data analysis was performed using STATA software (version 14.2; Stata Corp., College Station, TX, USA). Distribution normality and homogeneity of variance were assessed using the Shapiro-Wilk and Levene tests, respectively. To evaluate differences between the two groups while accommodating varying error distributions and controlling for potential confounding factors, Generalized Linear Models (GLzM) were employed. The GLMM framework was chosen because it unifies the analytical assumptions of ANOVA and ANCOVA within a flexible structure, allowing for the inclusion of continuous and categorical covariates through appropriate link functions without inflating Type I error rates. For all continuous variables, post-hoc pairwise comparisons were nested within the GLzM structure using the Dancan´s adjustment to account for multiple testing across the univariate outcomes. Results were presented as mean ± standard deviation or median with interquartile ranges, with a significance threshold set at α < 0.05.

### Clinical assessment and diagnosis of sarcopenia

## Results

Table 1 presents the characteristics of the sample. The elderly individuals in the Sarcopenic Group (ES) were significantly older, averaging 77.1 ± 10.8 years, compared to the Elderly Non-Sarcopenic Group (ENS), which averaged 73.8 ± 9.7 years (p < 0.05). Because of this age difference, all analyses were adjusted for age. There were no significant differences between the groups in terms of body mass index (BMI)—ES group: 26.3 ± 3.2 kg/m², ENS group: 27.7 ± 4.1 kg/m² appendicular skeletal muscle mass index (ASMI)—ES group: 6.6 ± 0.9 g/m², ENS group: 7.2 ± 2.5 g/m²—or body fat percentage—ES group: 39.8 ± 9.2%, ENS group: 40.2 ± 9.9% (p > 0.05). In terms of muscle function, the ES group had lower handgrip strength (21.7 ± 7.6 kg) compared to the ENS group (27.6 ± 10.4 kg; p < 0.05). Likewise, physical performance, as measured by the SPPB-Br, was lower in the ES group (8.8 ± 2.3 points) compared to the ENS group (11.2 ± 1.0 points; p < 0.05). Among the inflammatory biomarkers, only TNF-α levels were significantly higher in the ES group (4344.0 ± 394.2 pg/dL) than in the ENS group (4077.4 ± 400.0 pg/dL; p < 0.05). No significant differences were observed between the groups for the other biochemical and hormonal parameters. Regarding self-reported chronotype preference, most participants were classified as having a morning chronotype, representing 95.2% of the total sample. Only two participants were classified as intermediate chronotypes and one participant as an evening chronotype. No significant differences in chronotype scores were observed between groups.

**Table 1:** Sample characterization

Variables/Groups	ES		ENS	
	MD ± DP	(min–max)	MD ± DP	(min–max)
Age _(years)_	77.1 ± 10.8*	73.2–81.0	73.8 ± 9.7	70.3–77.3
BMI _(kg/m_^2^_)_	26.3 ± 3.2	25.1–27.5	27.7 ± 4.1	26.2–29.2
ASMI _(kg/m_^2^_)_	6.6 ± 0.9	6.3–6.9	7.2 ± 2.5	6.3–8.1
Body Fat _(%)_	39.8 ± 9.2	36.5–43.1	40.2 ± 9.9	36.6–43.8
Handgrip Strength _(kg)_	21.7 ± 7.6*	19.0–24.4	27.6 ± 10.4	23.8–31.4
SPPB-Br _(escore)_	8.8 ± 2.3*	8.0–9.6	11.2 ± 1.0	10.8–11.6
Glucose _(mg/dL)_	100.4 ± 21.6	92.6–108.2	100.4 ± 17.8	94.0–106.8
Triglycerides _(mg/dL)_	111.0 ± 50.0	93.0–129.1	122.7 ± 56.7	102.2–143.2
Total Cholesterol _(mg/dL)_	173.0 ± 48.4	155.5–190.5	189.2 ± 31.8	177.7–200.7
HDL _(mg/dL)_	57.8 ± 13.7	52.9–62.7	54.8 ± 12.9	50.1–59.5
LDL _(mg/dL)_	101.0 ± 32.2	89.4–112.6	109.8 ± 26.8	100.1–119.5
VLDL _(mg/dL)_	22.1 ± 8.2	19.1–25.1	24.6 ± 11.3	20.5–28.7
Vitamin D _(ng/dL)_	29.3 ± 9.2	26.0–32.6	27.8 ± 11.5	23.6–32.0
Albumin _(g/dL)_	4.2 ± 0.3	4.1–4.3	4.2 ± 0.3	4.1–4.3
Urea _(mg/dL)_	37.4 ± 10.7	33.5–41.3	38.6 ± 10.7	34 + 7–42.5
Creatinine _(mg/dL)_	3.6 ± 15.1	− 1.9–9.1	3.0 ± 12.9	-1.7–7.7
Cortisol _(µg/dL)_	11.1 ± 3.3	9.9–12.3	9.0 ± 2.2	8.2–9.8
Testosterone _(ng/dL)_	198.2 ± 264.7	102.6–293.8	170.6 ± 226.3	88.9–252.3
GH _(ng/dL)_	1.6 ± 1.9	0.9–2.3	1.5 ± 2.0	0.8–2.2
IGF-1 _(ng/dL)_	1.8 ± 1.5	1.3–2.3	1.5 ± 1.3	1.0–2.0
TNF-α _(pg/dL)_	4344.0 ± 394.2*	4201.7–4486.3	4077.4 ± 400.0	3933.0–4221.8
IL-6 _(pg/dL)_	29.1 ± 9.0	25.9–32.3	28.2 ± 8.9	25.0–31.4
IL-1ra _(ng/dL)_	0.9 ± 0.1	0.86–0.94	1.0 ± 0.3	0.9–1.1
IL-10 _(pg/dL)_	20.0 ± 5.6	18.0–22.0	19.1 ± 4.5	17.5–20.7

*GLzM *analyses

Data are expressed as mean ± standard deviation (*SD*), *BMI *= body mass index, *ASMI *= appendicular skeletal muscle mass index, %*BF *= body fat percentage, *SPPB-Br* = short physical performance battery

*g/dL* = grams per decilitre, Thou/mm³ = thousand units per cubic millimetre, mg/dL = milligrams per decilitre, % = percentage

*Different from the *ENS *group (*p* < 0.05)

Table 2 presents the results of the assessment of isokinetic and isometric peak torque. The absolute torque for thigh extension and flexion was significantly lower in the ES group, with values of 82.68 ± 35.06 N·m and 44.40 ± 23.74 N·m, respectively, compared to the ENS group, which had values of 113.00 ± 26.82 N·m for extension and 53.26 ± 15.99 N·m for flexion (*p* < 0.05 for both comparisons). Additionally, the ES group exhibited a lower relative thigh extension torque (128.13 ± 40.41%) compared to the ENS group (173.69 ± 69.93%; *p* < 0.05). Similarly, the relative thigh flexion torque was also lower in the ES group (68.55 ± 27.03%) than in the ENS group (82.36 ± 29.17%; *p* < 0.05). In terms of the agonist/antagonist relationship, the ES group showed a greater muscle imbalance (54.27 ± 15.61%) compared to the ENS group (47.22 ± 9.20%; *p* < 0.05). However, no significant differences were found between the two groups for the absolute and relative isometric torque parameters (*p* > 0.05).

**Table 2:** – Assessment of isokinetic and isometric peak torque

Variables/Groups	ES	ENS
Isokinetic peak torque - extension _(N.m)_	82.68 ± 35.06*	113.00 ± 26.82
Isokinetic peak torque - flexion _(N.m)_	44.40 ± 23.74*	53.26 ± 15.99
Relative isokinetic peak torque - extension _(%)_	128.13 ± 40.41*	173.69 ± 69.93
Relative isokinetic peak torque - flexion _(%)_	68.55 ± 27.03*	82.36 ± 29.17
Agonist/antagonist ratio _(%)_	54.27 ± 15.61*	47.22 ± 9.20
Isometric peak torque - extension (_N.m)_	102.37 ± 48.21	117.75 ± 32.86
Relative isometric peak torque - extension _(%)_	145.84 ± 53.44*	172.33 ± 62.44

*GLzM *analyses

Data presented in their respective scores and expressed as mean ± standard deviation, *N.m *= newton-meter (unit of torque), * Different from the *ENS *group; *p* < 0,05

Table 3 presents the results from the questionnaires that assessed depression, anxiety, and mood state. None of the groups exhibited a high depression profile (measured by the GDS) or anxiety trait (measured by the STAI TRAIT). According to the BRUMS, the vigor dimension was significantly lower in the ES group (8.79 ± 3.12 points) compared to the ENS group (11.30 ± 3.13 points; *p* < 0.05). Conversely, the Total Mood Disturbance (TMD) was higher in the ES group (0.12 ± 7.19 points) than in the ENS group (-2.11 ± 9.21 points; *p* < 0.05). No significant differences were observed between the groups for the other emotional states evaluated.

**Table 3:** – Questionnaires for depression anxiety trait and mood state assessment

Variables/Groups		ES	ENS	
Geriatric deppression scale (score)		2.16 ± 2.15	2.28 ± 2.42	
STAI trait (score)		41.18 ± 6.82	43.55 ± 6.89	
BRUMS (score)	Tension	2.33 ± 2.18		3.00 ± 3.08
	Depression	0.91 ± 1.28		0.88 ± 1.39
	Anger	1.12 ± 2.15		1.38 ± 2.28
	Vigor	8.79 ± 3.12*		11.30 ± 3.13
	Fatigue	1.66 ± 1.63		1.80 ± 2.02
	Confusion	1.45 ± 1.69		1.26 ± 1.51
	TMD	0.12 ± 7.19*		-2.11 ± 9.21

*GLzM *analyses

Data presented in their respective scores and expressed as mean ± standard deviation

* different from the *ENS *group; *p* < 0,05

Table 4 summarizes the sleep parameters assessed through polysomnography, actigraphy, and questionnaires. The total sleep time (TST) was significantly shorter in the ES group (307.7 ± 91.2 min) compared to the ENS group (351.9 ± 112.6 min; *p* < 0.05). Similarly, the Apnea-Hypopnea Index (AHI) was greater in the ES group (20.0 ± 17.9 events/hour) compared to the ENS group (10.4 ± 10.6 events/hour; *p* < 0.05).

In the actigraphy evaluation, the ES group showed lower sleep efficiency (90.0 ± 6.6%) compared to the ENS group (94.2 ± 4.1%; *p* < 0.05). Additionally, wakefulness after sleep onset (WASO) was longer in the ES group (35.4 ± 26.9 min) than in the ENS group (18.1 ± 12.1 min; *p* < 0.05). Due to technical issues with actigraphy data recording, only 19 records from the ES group and 21 from the ENS group were complete, precluding further analyses of sleep-wake regularity between the groups.

Regarding the subjective sleep questionnaires, the ES group presented higher ESS scores compared with the ENS group (8.5 ± 5.6 vs. 5.5 ± 3.7, respectively; *p* < 0.05). No significant differences were observed between the groups in sleep quality, sleep efficiency assessed by the Pittsburgh Sleep Quality Index (PSQI), or Insomnia Severity Index (ISI) scores (*p* > 0.05).

**Table 4:** – Sleep aspects assessment using overnight polysomnography, and questionnaires

		Variables/Groups		ES	ENS
Polysomnography	Sleep Latency _(minutes)_			25.6 ± 16.5	28.2 ± 26.5
	REM Sleep Latency _(minutes)_			104.6 ± 55.2	94.7 ± 54.5
	Total Sleep Time _(minutes)_			307.7 ± 91.2*	351.9 ± 112.6
	Sleep Efficiency _(%)_			68.3 ± 16.1	72.9 ± 9.2
	N1 _(%)_			15.8 ± 13.4	13.5 ± 7.9
	N2 _(%)_			38.9 ± 11.8	42.1 ± 12.6
	N3 _(%)_			28.3 ± 10.5	27.6 ± 9.5
	REM _(%)_			20.4 ± 12.2	18.4 ± 5.0
	WASO _(minutos)_			113.9 ± 55.2	97.9 ± 41.5
	AHI _(number/hour)_			19.9 ± 17.9*	10.4 ± 10.6
Actigraphy	Activity Time _(minutes)_			1004.0 ± 506.7	1029.5 ± 695.6
	Sleep Latency _(minutes)_			15.3 ± 19.3	9.2 ± 3.2
	Total Sleep Time _(minutes)_			251.6 ± 131.7	271.0 ± 118.9
	Sleep Efficiency _(%)_			89.9 ± 6.6*	94.2 ± 4.0
	WASO _(minutes)_			35.3 ± 26.9*	18.1 ± 12.1
Questionnaires			Epworth _(daytime sleepness - score)_	8.5 ± 5.6*	5.5 ± 3.6
			ISI _(score)_	6.1 ± 4.1	7.8 ± 4.8
			PSQI _(sleep quality - score)_	5.0 ± 2.4	6.4 ± 3.8
			PSQI _(sleep efficiency %)_	81.0 ± 14.8	80.8 ± 15.5

GLzM analyses

Data are expressed as mean ± standard deviation (SD), REM = rapid eye movement sleep, N1 = non-rapid eye movement sleep stage 1, N2 = non-rapid eye movement sleep stage 2, N3 = non-rapid eye movement sleep stage 3, WASO = wake after sleep onset,* AHI = *apnea-hypopnea index, PSQI = pittsburgh sleep quality index, ISI = Insomnia severity index

*Different from the *ENS *group (p < 0.05)

## Discussion

The findings of the present study indicate that sarcopenic older adults exhibited shorter total sleep time during polysomnographic assessment, lower sleep efficiency and longer wakefulness after sleep onset as assessed by actigraphy, and a higher apnea-hypopnea index and circulating TNF-α levels compared with non-sarcopenic older adults. In the present sample, sarcopenic participants were older than non-sarcopenic participants, a finding that is consistent with previous evidence showing an increased prevalence of sarcopenia with advancing age [25]. Even after adjusting for age, the appendicular skeletal muscle mass index (ASMI) remained similar between groups, whereas handgrip strength and physical performance were lower in sarcopenic participants. Although sarcopenia was diagnosed according to the original EWGSOP criteria, these findings are consistent with subsequent evidence indicating that muscle strength and physical performance are more strongly associated with adverse outcomes than muscle mass alone [26].

No differences were observed in depressive symptoms or anxiety traits between groups. However, sarcopenic older adults presented lower vigor and higher TMD scores. The reduction in vigor may reflect poorer physical performance and muscle function, leading individuals with sarcopenia to perceive lower motivation and energy for daily activities [27, 28]. Because all questionnaires were administered at the same time of day, the influence of circadian factors on mood perception was minimized. Nevertheless, these findings should be interpreted cautiously because the Horne and Östberg questionnaire assesses self-reported chronotype preference rather than objective circadian phase. This finding should be interpreted with caution, as the Horne and Östberg questionnaire assesses self-reported chronotype preference and does not provide an objective measure of circadian phase.

In this context, the present findings suggest that sarcopenic older adults experience more compromised sleep patterns compared with non-sarcopenic individuals, including shorter total sleep time during polysomnography and actigraphy-derived evidence of lower sleep efficiency and longer wakefulness after sleep onset. Although total sleep time differed between groups during polysomnography, no significant differences were observed in actigraphy-derived total sleep time over the monitoring period. Therefore, the reduced sleep duration observed during polysomnography should be interpreted cautiously and may reflect night-to-night variability or the laboratory sleep environment rather than habitual sleep duration. These results are consistent with studies showing that sleep pattern and quality are linked to sarcopenia risk. One study reported that inadequate sleep behaviors in older adults increase the likelihood of muscle function decline, indicating that better sleep habits may help preserve muscle health during aging [29]. Another study found that older adults with poor sleep quality present a higher prevalence of sarcopenia, reinforcing the importance of sleep for musculoskeletal integrity [30].

A higher apnea-hypopnea index (AHI) was observed among sarcopenic individuals. This finding aligns with evidence suggesting that sleep-disordered breathing may contribute to the development of sarcopenia and sarcopenic obesity through mechanisms involving chronic inflammation, metabolic imbalance, and vitamin D deficiency [18]. In addition, older adults with sleep-disordered breathing have been shown to exhibit a greater likelihood of sarcopenia and osteoporosis, highlighting broader musculoskeletal impacts associated with these sleep disturbances [31].

The literature also indicates that both insomnia and obstructive sleep apnea (OSA) are associated with excessive daytime sleepiness and poorer sleep quality [13, 32]. Although subjective sleep quality did not differ between groups, the non-sarcopenic group presented numerically higher PSQI scores, suggesting that subjective sleep perception may not necessarily parallel objective sleep impairments. In the present sample, both groups exhibited relatively poor self-reported sleep quality, indicating that sleep complaints are common among older adults regardless of sarcopenia status. Conversely, older adults with sarcopenia exhibited higher scores on the Epworth Sleepiness Scale (ESS) than those without sarcopenia. It is important to note, however, that these findings indicate a greater self-reported propensity to fall asleep in everyday situations, as assessed by the ESS, rather than providing a direct measure of daytime sleepiness [33, 34]. Given the multifactorial nature of sleepiness-related responses, the present study cannot distinguish the potential contributions of inflammation, the severity of sleep-disordered breathing, and other factors associated with aging. Therefore, these results should be interpreted with caution, without inferring a direct causal relationship.

The increase in inflammatory markers and the sleep alterations observed in sarcopenic older adults suggest the existence of a vicious cycle involving aging, chronic inflammation, and muscle impairment. Evidence indicates that these inflammatory pathways contribute not only to muscle degradation but also to the deterioration of sleep parameters such as latency, fatigue, and daytime sleepiness, as well as to metabolic and cardiovascular alterations [13, 35], which, in turn, may further aggravate muscle loss. A recent meta-analysis demonstrated that individuals with sarcopenia exhibit significantly higher levels of the systemic immune-inflammation index (SII), reinforcing the role of chronic inflammation in the pathophysiology of sarcopenia [31]. It is worth noting that the elevation of pro-inflammatory cytokines, such as TNF-α, can trigger a cascade of molecular events involving the activation of nuclear factor κB (NF-κB), which may lead to a series of mitochondrial dysfunctions [36]. These dysfunctions can result in reduced phosphorylation of the AKT-mTOR pathway, thereby diminishing protein synthesis signaling [37, 38], as well as enhancing the activation of proteolytic pathways such as the ubiquitin-proteasome system [39]. This molecular environment, characterized by persistent inflammation as observed in sarcopenia along with mitochondrial impairment and inhibition of anabolic signaling, creates favorable conditions for muscle atrophy, particularly affecting glycolytic fibers, which are most susceptible to sarcopenia [40–42].

These results corroborate other findings demonstrated by our group that highlight the importance of interventions targeting both sleep quality and systemic inflammation as strategies to slow the progression of sarcopenia [19–43]. On the other hand, cohort studies or longer clinical trials are essential to confirm these associations. In addition, technical issues with actigraphy led to the loss of some data, preventing a more comprehensive analysis of the sleep-wake cycle and its relationship with physical and metabolic outcomes. The absence of complete data on anabolic and catabolic hormone secretion (e.g., testosterone, GH, and IGF-1) also restricted the interpretation of physiological mechanisms involved in sarcopenia and their potential connection to sleep quality.

This study has some limitations that should be acknowledged. The relatively small sample size and participant attrition may have limited the statistical power to detect more subtle associations between sleep, inflammation, and sarcopenia. In addition, technical issues with actigraphy resulted in incomplete recordings, preventing a more comprehensive assessment of sleep-wake patterns. The limited hormonal characterization and the unavailability of individual polysomnographic and oximetry data also restricted further exploration of the mechanisms linking sleep disturbances, nocturnal hypoxemia, inflammation, and sarcopenia. Therefore, the potential contribution of nocturnal hypoxemia to inflammatory status and sarcopenia could not be fully explored. Finally, although the inflammatory biomarkers evaluated in this study are among the most relevant indicators of inflammatory balance, additional approaches involving immune cell stimulation may provide a more comprehensive understanding of the relationship between sleep and sarcopenia.

Future studies with larger samples, more detailed analyses of the sleep-wake cycle, and the inclusion of additional hormonal and inflammatory biomarkers may contribute to a better understanding of the relationship between sleep and sarcopenia in older adults.

## Conclusions

This study demonstrated that sarcopenic older adults not only exhibit poorer physical performance and reduced muscle strength but also present distinct alterations in sleep patterns, including shorter total sleep time during polysomnographic assessment, lower sleep efficiency and longer wakefulness after sleep onset as assessed by actigraphy, and a higher apnea-hypopnea index. These individuals also presented higher scores on the Epworth Sleepiness Scale than non-sarcopenic participants. Such alterations were accompanied by higher TNF-α levels in the sarcopenic group, indicating a pattern consistent with heightened inflammatory activity. Together, these findings suggest that sleep disturbances and inflammation may represent important targets for future strategies aimed at understanding and potentially mitigating the progression of sarcopenia.

## Data Availability

The data will be shared on reasonable request to the corresponding author.
